# Endocrine manifestations in adults with 22q11.2 deletion syndrome: a retrospective single-center cohort study

**DOI:** 10.1007/s40618-023-02276-0

**Published:** 2024-02-03

**Authors:** E. Soubry, K. David, A. Swillen, E. Vergaelen, M. Docx Op de Beeck, M. Hulsmans, S. Charleer, B. Decallonne

**Affiliations:** 1 Department of Endocrinology, University Hospitals Leuven, Louvain, Belgium; 2 Department of Genetics, University Hospitals Leuven, Louvain, Belgium; 3 Department of Psychiatry, University Hospitals Leuven, Louvain, Belgium

**Keywords:** 22q11DS, Hypoparathyroidism, Thyroid disease, Obesity

## Abstract

**Introduction and objective:**

Patients with the 22q11.2 deletion syndrome (22q11DS) frequently display cardiological and psychiatric diseases, but are also at increased risk for endocrine manifestations. The aim of this study was to evaluate the screening, prevalence, and management of hypoparathyroidism and thyroid disease in patients with 22q11DS, to evaluate the metabolic profile, and to compare these results with current literature and guidelines.

**Design:**

We performed a retrospective study of patients with genetically confirmed 22q11DS, followed at the center for human genetics of the University Hospitals Leuven, resulting in a cohort of 75 patients. Medical history, medication, and laboratory results concerning hypoparathyroidism, thyroid dysfunction, and the metabolic profile were collected.

**Results:**

Of the total cohort, 26 patients (35%) had at least one hypocalcaemic episode. During hypocalcaemia, parathyroid hormone (PTH) was measured in only 12 patients with 11 having normal or low PTH, confirming a diagnosis of hypoparathyroidism. Recurrent episodes of hypocalcaemia occurred in seventeen patients (23%). Adherence to the guidelines was low, with 13% of patients having a yearly serum calcium evaluation, 12% receiving daily calcium supplements, and 20% receiving non-active vitamin D. Hypothyroidism was present in 31 patients (44%) and hyperthyroidism in 6 patients (8%). Information on body mass index (BMI) was available in 52 patients (69%), of which 38% were obese (BMI ≥ 30 kg/m^2^).

**Conclusion:**

Hypoparathyroidism, hypothyroidism, and obesity are common endocrine manifestations in patients with 22q11DS but are probably underdiagnosed and undertreated, indicating the need for multidisciplinary follow-up including an endocrinologist.

## Introduction

The 22q11.2 deletion syndrome (22q11DS), which is also referred to as DiGeorge syndrome or velocardiofacial syndrome, is a genetic disorder with autosomal dominant inheritance occurring in approximately 1/4000 to 1/9000 births. The prevalence is rising over time because of two main reasons. First, there is more detection of the syndrome. Second, the survival of children with 22q11DS is higher due to better treatment for congenital cardiac disorders, resulting in more patients reaching adult age and potentially transmitting the deletion to their children. Due to the aforementioned autosomal dominant inheritance, half of the children of affected parents will have the deletion [1, 2]. The 22q11DS has a highly variable clinical phenotype. Possible clinical features consist of congenital cardiac defects, endocrine disorders, thymic hypoplasia, velopharyngeal insufficiency, developmental delay, and psychiatric disorders [2–6].

The most frequent endocrine manifestation associated with 22q11DS is hypocalcaemia due to hypoparathyroidism [5–7]. The prevalence of hypocalcaemia in 22q11DS varies highly among different studies, ranging from 30 to 80% [8]. Hypoparathyroidism is considered the most important cause of hypocalcaemia in 22q11DS and is secondary to congenital parathyroid aplasia/hypoplasia [9]. The presentation of hypoparathyroidism in 22q11DS ranges from intermittent hypocalcaemia during elevated stress (e.g., neonatal period, surgery) to chronic mostly mild hypocalcaemia. Depending on the severity and the acuteness of onset of hypocalcaemia, affected patients can experience a wide range of symptoms and signs, such as seizures, laryngospasms, paraesthesia, ‘brain fog’, and prolonged QT, or be completely asymptomatic [10, 11]. Conventional treatment of hypoparathyroidism consists of calcium supplements, non-active (e.g., cholecalciferol) and active vitamin D (e.g., calcitriol or calcifediol). Additionally, anti-calciuretic drugs (e.g., thiazide diuretics) may be used, especially when hypercalciuria is present [12]. Recombinant human PTH 1-84 has been FDA- and EMA-approved for use in patients with hypoparathyroidism not well controlled by conventional therapy, but this treatment is unavailable in many countries (such as Belgium) [12]. New drugs, such as long-acting PTH analogs, are emerging [13]. Over time, both hypoparathyroidism and its treatment may result in ectopic soft tissue calcifications in the brain (mainly in the basal ganglia), the eyes (cataracts) and the kidneys (stones and nephrocalcinosis), which has been attributed to an elevated calcium-phosphate product and chronic hypercalciuria, in particular under treatment with vitamin D analogs. Therefore, guidelines advise us to monitor serum calcium at least twice yearly and 24-h calciuria once yearly, to perform an ophthalmic exam at least at baseline, to perform renal and brain imaging at least at baseline, and to repeat renal imaging every 5 years [10–12, 14]. Besides hypoparathyroidism, an increased prevalence of functional and structural thyroid diseases has been observed. Both a higher prevalence of auto-immune hypothyroidism and Graves’ hyperthyroidism have been reported [15, 16]. Current knowledge on both parathyroid and thyroid disorders in patients with 22q11DS is however limited and mainly derived from case reports and small retrospective studies [7, 9, 15–17]. Finally, higher prevalence of obesity among patients with 22q11DS has also been reported [18].

The aim of this study was to evaluate the screening, prevalence, and management of hypoparathyroidism and thyroid disease, and to investigate the metabolic profile in a cohort of patients with 22q11DS and to compare these results with current literature and guidelines for endocrine management in 22q11DS patients.

## Methods

### Patient identification

This is a monocentric sub-study of patients included in a multi-centric study on psychiatric disorders in 22q11 DS (International Brain Behaviour Consortium on 22q11.2 deletion syndrome [19]). The cohort included in this study consists of adult patients with 22q11.2 deletion syndrome followed at center for human genetics of the University Hospitals of Leuven, Belgium, a tertiary care center, between 1 January 2013 and 31 December 2018. The patients were studied by detailed review of their medical records. The 22q11.2 deletion syndrome was confirmed by standard fluorescence in situ hybridization or genome-wide microarray techniques. The study was approved by the Ethical Committee of the University Hospital of Leuven (S-number S52418). All patients or their legal representatives gave written consent to participate to the study.

### Data collection

Patient characteristics, such as sex, age, age at genetic diagnosis, length, and body weight, at the most recent clinical visit were retrieved from the electronic medical records before 1 April 2022. Medical history was reviewed in detail for seizures, epilepsy, thyroid and parathyroid disorders, diabetes mellitus, arterial hypertension, dyslipidemia, liver steatosis, obesity, and congenital cardiac and psychiatric disorders. Current use of the following medication was registered: calcium supplements, vitamin D supplements, active vitamin D analogs, levothyroxine, anti-thyroid drugs, antihypertensive drugs, statins, and antidiabetic drugs. The following lifetime biochemical data (meaning any available data between birth and April 2022) on calcium and thyroid homeostasis were collected: total serum calcium, albumin, phosphate, magnesium, parathyroid hormone (PTH), creatinine, calculated eGFR (CKD-EPI formula), thyroid stimulating hormone (TSH), free T4, free T3, TPO-Abs, TSHR-Abs and 24-h calciuria. Due to the retrospective nature of this observational study, there was no standardized way in which biochemistry was assessed, and data were collected whenever they were available. Additionally, the following most recently available biochemical parameters were registered: 25OH vitamin D, gamma-glutamyltransferase (gGT), aspartate aminotransferase (AST), alanine aminotransferase (ALT), glycated hemoglobin (HbA1c), fasting glycemia, total cholesterol, high-density lipoprotein (HDL), low-density lipoprotein (LDL), and triglycerides. Furthermore, the reports of any imaging of the kidneys (ultrasonography (US) or computed tomography (CT)), liver (US/CT), thyroid gland (US), and brain (CT or magnetic resonance imaging (MRI)) performed since birth were analyzed.

### Definitions

Hypocalcaemia was defined as a total serum calcium < 2.1 mmol/L [20, 21]. Hypoparathyroidism was defined as either the biochemical finding of hypocalcaemia in combination with a low or inadequately normal PTH, or based on the diagnosis of hypoparathyroidism mentioned in the medical history, or by the use of active vitamin D except for other indications (e.g., post-operative hypoparathyroidism, chronic kidney disease).

Hypothyroidism was either defined as a serum TSH > 5 mIU/L in the absence of anti-thyroid drug use, or based on the diagnosis of hypothyroidism mentioned in the medical history, or by the use of levothyroxine. Hyperthyroidism was defined as a TSH < 0.4 mIU/L. Underlying thyroid disease etiology was based on presence of TPO-Abs, TSH-R Abs, and US findings. Diabetes mellitus was defined as a HbA1c > 6.5% on two different occasions [22], or by the use of antidiabetic drugs. Dyslipidemia was defined as a total non-fasting cholesterol of > 190 mg/dL and/or an LDL-cholesterol > 115 mg/dL [23]. Liver steatosis was defined as steatosis mentioned in the US report or a non-alcoholic fatty liver fibrosis (NALFD) score > − 1.5 [24].

### Statistical analysis

Continuous data are presented as median [interquartile range]. Categorical data are presented as percentages (% of n). For the analysis of categorical data, we used the Pearson’s *X*^2^-test via the statistical program SAS/SPSS for Windows (IBM SPSS Statistics version 23, Armonk, USA). A two-tailed *p* value < 0.05 was considered as statistically significant.

## Results

### Patient characteristics

In total, 75 patients were included in the study with 43% males and 57% females. The median age was 30 years [interquartile range 25–36]. The median age at genetic confirmation of 22q11.2DS was 6 years [min–max range 0–32]. At least one congenital heart abnormality was present in 60% of the patients, with ventricular septal defect in 28%, pulmonary valve stenosis in 27%, tetralogy of Fallot in 24%, atrial septum defect in 11%, and one patient with malposition of the large vessels. At least one psychiatric disorder was present in 70% of the patients, with depression in 47%, psychosis in 29%, autism spectrum disorder in 24%, and attention-deficit hyperactivity disorder in 7%. Mental retardation (IQ < 70) was present in 32% of the patients.

### Hypocalcaemia and hypoparathyroidism

#### Screening, prevalence, and monitoring

**Fig. 1:**
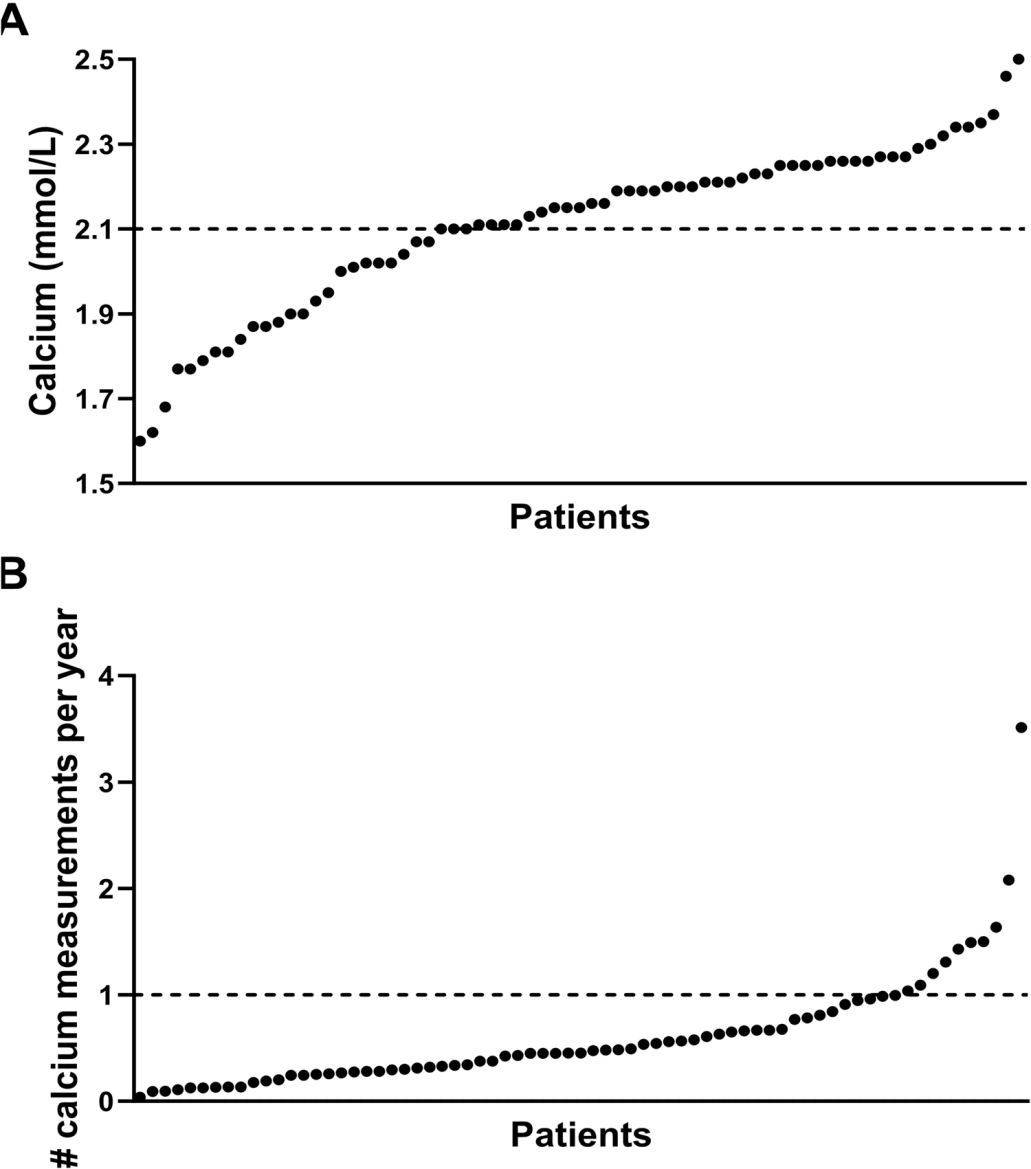
**A** Lowest serum calcium (mmol/L) per patient **B** median number of serum calcium measurements per year per patient. Each dot represents 1 individual patient (*n* = 71). The horizontal line represents the cut-off value of 2.1 mmol/L (**A**) and the cut-off of a yearly calcium measurement (**B**). Figures made by GraphPad Prism v10.0.0 (La Jolla, USA)

As shown in Table 1, during lifetime, at least one serum calcium and PTH was determined in 95% and 69% of the patients, respectively. Hypocalcaemia was documented in 26 patients (35%), with 2 patients presenting with neonatal hypocalcaemia. Serum calcium during a hypocalcaemic episode ranged from 1.60 to 2.10 mmol/L, with a median of 1.99 mmol/L (Fig. 1A). PTH was measured during the hypocalcaemic episode in 12 patients (46%), with 11 patients having a normal or low PTH and 1 patient with elevated PTH level. Of all patients, 31 (41%) had at least one consultation with an endocrinologist of which 9 (29%) were diagnosed with hypoparathyroidism (based on medical history). The median number of serum calcium measurements per year was 0.45, and only 10 patients (13%) had a yearly measurement of serum calcium levels (Fig. 1B) The proportion of patients with hypocalcaemia suffering from congenital heart disease was not different from those without hypocalcaemia (65% vs. 57%, *p* = 0.448).

**Table 1:** Hypoparathyroidism: screening, prevalence, follow-up

	*n* = 75
*Screening*	
≥ 1 serum calcium measurement performed	71/75 (95)
≥ 1 serum PTH measurement performed	52/75 (69)
*Prevalence*	
Patients with ≥ 1 low serum calcium	26/75 (35)
Low/normal PTH during hypocalcaemia	11/12 (46)
Hypoparathyroidism based on biochemical data	11/75 (15)
Hypoparathyroidism mentioned in medical history	10/75 (13)
*Follow-up*	
At least 1 consultation with endocrinologist	31/75 (41)
Yearly calcium measurement	10/75 (13)
% with endocrinologist consultation	6/10
% with hypoparathyroidism	5/10

Data are presented as *n* (%) for categorical data

*PTH* parathyroid hormone

#### Treatment and complications

As shown in Table 2, 9 patients (12%) of the total cohort were currently taking calcium supplements and 15 patients (20%) were currently taking cholecalciferol. Within the subgroup with diagnosis of hypoparathyroidism reported in the medical history, 80% was taking calcium and 80% was taking cholecalciferol, whereas only 1 patient (1%) and 7 patients (10%) of the subgroup without diagnosis of hypoparathyroidism were taking calcium and cholecalciferol, respectively. All 6 patients on active vitamin D had a confirmed diagnosis of hypoparathyroidism and were concomitantly taking calcium supplements. Only 5 of 6 were also concomitantly taking non-active vitamin D.

**Table 2:** Hypoparathyroidism: treatment, acute and chronic complications

	*n* = 75
*Medical treatment*	
Calcium carbonate	9/75 (12)
Subgroup with hypoparathyroidism	8/10 (80)
Subgroup without hypoparathyroidism	1/65 (1)
Cholecalciferol	15/75 (20)
Subgroup with hypoparathyroidism	8/10 (80)
Subgroup without hypoparathyroidism	7/65 (10)
Active vitamin D	6/75 (8)
Subgroup with hypoparathyroidism	6/10 (60)
Calcium carbonate and/or cholecalciferol and/or active vitamin D	16/75 (21)
% with hypoparathyroidism	9/16
Recurrent hypocalcaemia under treatment in hypoparathyroidism subgroup	6/9
*Acute complications*	
≥ 2 hypocalcaemic episodes	17/75 (23)
≥ 3 hypocalcaemic episodes	10/75 (13)
Hospitalization due to hypocalcaemic episodes	1/75 (1)
Convulsions due to hypocalcaemia	1/75 (1)
*Chronic complications*	
Kidneys	
≥ 1 imaging (US/CT) performed	48/75 (64)
Presence of nephrocalcinosis or lithiasis at imaging	1/48
Brain	
≥ 1 imaging (CT/MRI) performed	25/75 (33)
Presence of cerebral calcification	2/25

Data are presented as *n* (%) for categorical data

*US* ultrasonography, *CT* computed tomography, *MRI* magnetic resonance imaging

Concerning acute complications, 23% of the total cohort were having recurrent hypocalcaemic episodes. Only one patient was hospitalized due to hypocalcaemia, which presented as convulsions at neonatal age. On the other hand, 30 (35%) out of all the hypocalcaemic episodes (*n* = 84) occurred during a hospitalization for other reasons.

Chronic complications of hypoparathyroidism and its treatment concern ectopic calcifications. Renal imaging was performed in 48 patients (64%), in most cases for other indications than screening for nephrocalcinosis or lithiasis. Within the subgroup with hypoparathyroidism reported in the medical history, renal imaging was present in 9 out of 10 patients with one patient having renal calcinosis. Imaging of the brain was performed in 25 patients (33%), again mostly for other indications than screening for brain calcifications. Seven out of 10 patients with diagnosis of hypoparathyroidism mentioned in the medical history had at least one CT/MRI of the brain. Two patients, under active vitamin D treatment, had signs of brain calcifications.

### Thyroid disease

**Fig. 2:**
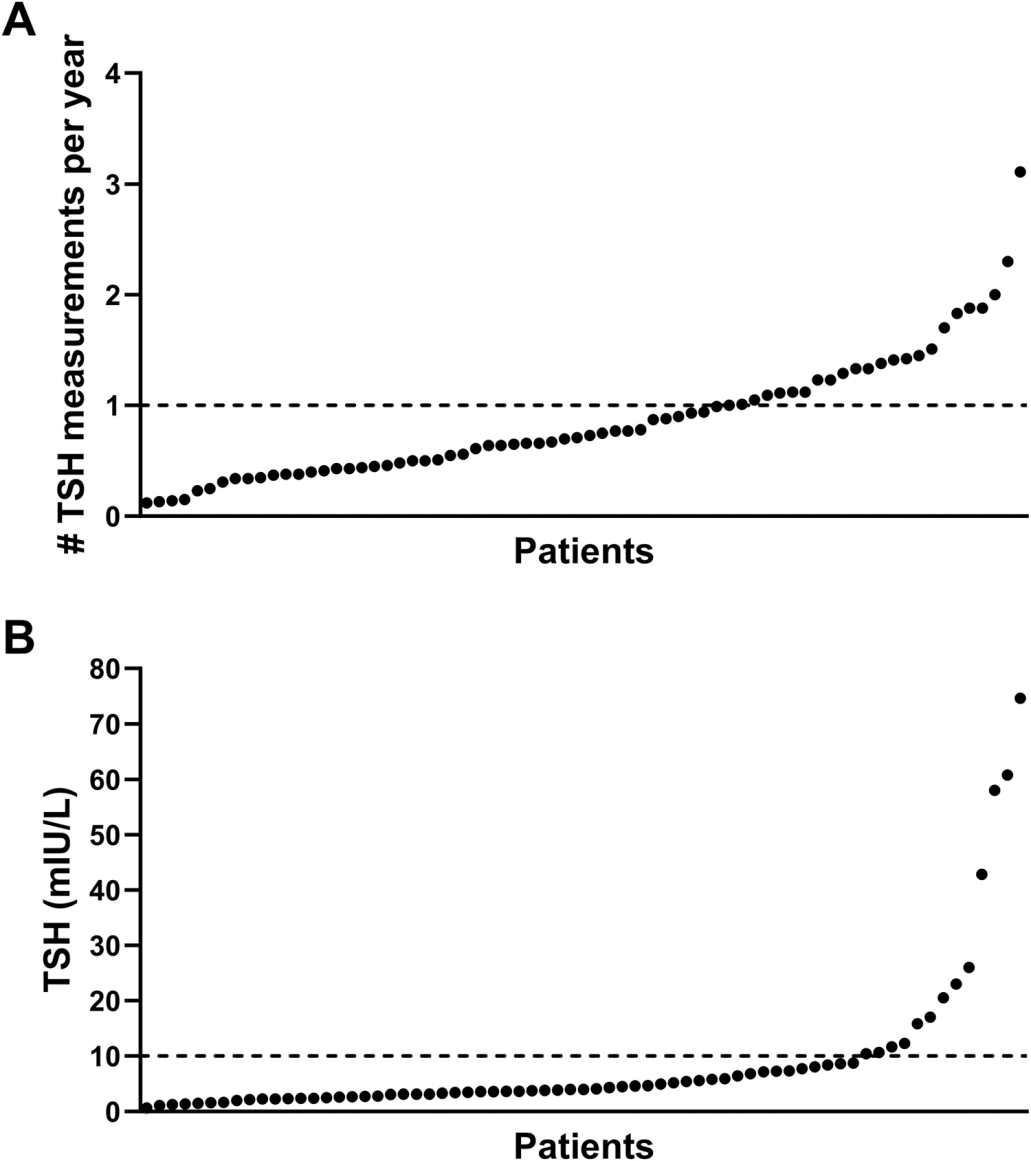
**A** Number of serum TSH measurements per year per patient **B** highest TSH (mIU/L) per patient (one patient had highest TSH 438 mIU/L and is not shown in the figure). Every dot represents 1 individual patients (*n* = 69). The horizontal line represents the cut-off of a yearly TSH measurement (**A**) and the cut-off value of TSH 5 mIU/L (**B**). Figures made by GraphPad Prism v10.0.0 (La Jolla, USA)

Findings related to screening and prevalence of thyroid disease are summarized in Table 3. At least one TSH measurement could be retrieved in 70 patients (93%). The median number of TSH measurements per year was 0.72, ranging from 0.12 to 3.11 per year. In 24 patients (32%), TSH was measured at least once a year (Fig. 2A).

**Table 3:** Thyroid disease: screening, prevalence of functional and structural disease

	*n* = 75
**Functional thyroid disease**	
*Screening*	
≥ 1 serum TSH performed	70/75 (93)
*Prevalence*	
Hypothyroidism	
Diagnosis mentioned in medical history	26/75 (35)
≥ 1 TSH measurement > 10 mIU/L	14/70 (20)
≥ 1 TSH measurement > 5 mIU/L	31/70 (44)
≥ 2 TSH measurement > 5 mIU/L	26/70 (37)
TPO-Abs positive	7/31
TPO-Abs negative	7/31
TPO-Abs not performed	17/31
Hyperthyroidism	
Diagnosis mentioned in medical history	2/75 (3)
≥ 1 TSH measurement < 0.4 mIU/L	14/70 (20)
TSHR-Abs positive	2/14
TSHR-Abs negative	2/14
TSHR-Abs not performed	2/14
Other etiology	8/14
**Structural thyroid disease**	
*Screening*	
≥ 1 thyroid US performed	12/75 (16)
*Prevalence*	
Ectopic lingual thyroid (diagnosis mentioned in medical history)	1/75 (1)
Thyroid nodule (diagnosis mentioned in medical history)	2/75 (3)
Thyroid nodule (non-suspect) at US	3/12
Thyroiditis at US	6/12
No abnormalities at US	3/12

Data are presented as n (%) for categorical data

*TSH* thyroid stimulating hormone, *TPO-Abs* thyroid peroxidase antibodies, *TSHR-Abs* TSH-receptor antibodies, *US* ultrasonography

Hypothyroidism was mentioned in the medical history in 26 patients (35%), which was confirmed by a TSH > 5 mIU/L on at least 2 blood samples. 31 patients (44%) had at least one TSH > 5mIU/L and 14 patients (20%) had a TSH > 10 mIU/L (Fig. 2B). TPO-Abs were positive in 7 (22%) and negative in 7 (22%) patients. In the remaining 17 cases of hypothyroidism, no measurement of TPO-Abs could be found. In 2 patients, hypothyroidism was caused by treatment with amiodarone and thyroidectomy for Graves’ disease, respectively.

In 14 patients (20%), TSH was < 0.4 mIU/L with 3 patients having a mildly decreased TSH during hospitalization attributed to non-thyroidal illness and 5 patients having a transiently low TSH due to oversupplementation with levothyroxine. The remaining 6 patients (8%) with TSH < 0.4 mIU/L were compatible with having a diagnosis of hyperthyroidism. TSHR-Abs were measured in only 4 patients with low TSH and were positive in 2 patients.

With regard to structural thyroid disease, one patient had an ectopic lingual thyroid gland diagnosed with CT scan of the head/neck region. Thyroid US was performed in 12 patients (16%). There were no cases of thyroid hypoplasia.

In total, 20 patients (27%) were taking levothyroxine, of which 18 had documented hypothyroidism. The other 8 patients with documented hypothyroidism were not receiving levothyroxine. Seven untreated patients experienced multiple episodes of hypothyroidism (based on biochemical data), not linked to a hospitalization.

### Metabolic profile

As shown in Table 4, a recent BMI was available in 52 patients (69%) with 35 (67%) of them being overweight (BMI ≥ 25 kg/m^2^) and 20 patients (38%) being obese (BMI ≥ 30 kg/m^2^). Four patients (5%) underwent bariatric surgery. Within the subgroup with BMI ≥ 30 kg/m^2^, 50% were treated with antipsychotic drugs, which did not statistical significantly differ from patients with BMI < 30 kg/m^2^ (59%) (*p* = 0.595). In contrast, hypothyroidism (TSH > 5mIU/L) was more frequent in patients with BMI ≥ 30 kg/m^2^ compared to non-obese patients (75% vs. 38%, *p* = 0.011). The presence of metabolic complications is shown in Table 4.

**Table 4:** Metabolic profile

	*n* = 75
*Screening for obesity*	
≥ 1 recent BMI available	52/75 (69)
BMI ≤ 25	17/52 (33)
BMI 25–29	15/52 (29)
BMI 30–35	12/52 (23)
BMI ≥ 35	8/52 (15)
*Prevalence of other metabolic conditions*	
Diabetes mellitus type 2	4/75 (5)
Hypertension	10/75 (13)
Dyslipidemia	25/75 (33)
*Screening for liver steatosis*	
≥ 1 US of the liver performed	35/75 (47)
Presence of Liver steatosis	6/35 (17)
≥ 1 NAFLD score available	37/75 (49)
NAFLD score > − 1.5	15/37 (40)
*Use of medication related to metabolic syndrome*	
Antidiabetics	4/73 (5)
ACEi/ARB	4/73 (5)
CCB	1/73 (1)
B-blocker	9/73 (12)
Statin	5/73 (7)
Antipsychotics	39/73 (53)
% with BMI > 30 kg/m^2^	10/20 (50)
*Bariatric surgery*	4/75 (5)

Data are presented as *n* (%)

*ACEi* angiotensin-converting enzyme inhibitor, *ARB* angiotensin II receptor blocker, *B-blocker* beta-blocker, *BMI* body mass index, *CCB* calcium channel blocker, *NAFLD score* non-alcoholic fatty liver fibrosis score, *US* ultrasonography

## Discussion

This retrospective study analyzed the screening, prevalence, treatment, and complications of parathyroid and thyroid disorders in a cohort of patients with 22q11 DS in follow-up at a tertiary outpatient clinic in Belgium. Our cohort consists of young adult patients (age range 20–55y), consistent with other cohorts [8, 18, 25, 26]. The prevalence of congenital heart defects in our cohort (60%) is also in agreement with others [8, 15, 17, 18, 27]. Only one-third of the studied cohort had a mental retardation, whereas in literature 40–50% of patients with 22q11DS has a mental retardation. [28, 29] The better performance status of our cohort can be explained by selection bias due to the inclusion procedure in the International Brain Behaviour Study [19]; in this study, patients needed to be able to complete a thorough psychiatric interview.

The prevalence of hypocalcaemia in patients with 22q11 DS in the literature varies highly which is at least partially explained by different definitions used for hypocalcaemia and different patient populations studied. In the study of Cheung et al*.*, 80% of patients suffered from hypocalcaemia, defined as an ionized calcium < 1.2 mmol/L [8]. In contrast, a prevalence of only about 30% was reported when using total calcium levels < 2.2 mmol/L for defining hypocalcaemia [9, 17]. The largest study to date investigating hypocalcaemia in 22q11 DS by Ryan et al. showed an intermediate prevalence of 60%, though no clear definition for hypocalcaemia was provided [3, 26]. In our cohort, 35% of patients had at least one hypocalcaemic episode defined as a total serum calcium < 2.10 mmol/L. Hypocalcaemia according to the guidelines is defined as an albumin-corrected serum calcium below the lower reference value, ranging from < 2.10 to < 2.20 mmol/L, depending on the assay [10, 20, 21]. In our study, we used the total serum calcium, because serum albumin was not available in the majority of cases. Since our cohort consists of a young population, we assumed that the serum albumin would be within normal range in most cases. As in previous studies, hypocalcaemia in our cohort was often recurrent. Data on PTH-levels in patients with 22q11 DS are even more scarce. The presence of low PTH again varies highly in literature, from 13 to 63% [8, 17, 27]. In our cohort, we had information on PTH at the time of hypocalcaemia in less than half of the patients. Diagnosis of hypoparathyroidism was mentioned in the medical history in only 13% of patients, in contrast to hypocalcaemia being detected in 35% and recurrent hypocalcaemia in 20% of patients, which might suggest underdiagnosis.

The higher prevalence of thyroid disorders reported in patients with 22q11 DS mainly concerns a higher prevalence of primary hypothyroidism. The prevalence of hypothyroidism agrees with previous studies with prevalence ranging between 20 and 30% [8, 15, 26, 31]. Several case reports suggest a higher prevalence of autoimmune hyperthyroidism, but larger studies to support this finding are lacking [32–34]. Besides abnormalities in thyroid function, previous studies also reported a higher prevalence of structural thyroid diseases. De Almeida et al. showed a prevalence of nodules in 50%, Stagi et al*.* in 16% of the patients [15, 16]. In this cohort, the prevalence of structural thyroid disease was only 5%. Autopsy studies suggest increased finding of abnormal thyroid morphology (e.g., lobe hypoplasia or lack of isthmus) [34]. In this study, one patient had an ectopic lingual thyroid, otherwise no thyroid hypoplasia could be diagnosed, potentially due to the low execution of thyroid US.

More than one-third of this cohort was obese (BMI ≥ 30 kg/m^2^)^2^. Voll et al. performed a large retrospective cohort study on adults with 22q11DS (*n* = 202) and reported a BMI ≥ 30 kg/m^2^ in 43% of patients [18], which is consistent with our observation. The high use of psychotropic medication in this population, as well as the high prevalence of congenital heart disease, could contribute to the higher prevalence of obesity. In the study of Voll et al., use of psychotropic medication was significantly associated with obesity [18], which could not be confirmed in our study. TSH levels > 5mIU/L were more prevalent in obese compared to non-obese patients. The reason for this however is complex, because an elevated TSH could be both cause and result of obesity [35]. Early growth abnormalities including being small for gestational age are other possible contributing factors to adult obesity in a population with 22q11 DS [36]. In our cohort, 5% of the patients had type 2 diabetes mellitus, which is consistent with the study by Voll et al. [18]. This may be an underestimation due to the relatively young age in both studies. Furthermore, one-third of our cohort had dyslipidemia, whereas less than 10% was treated with lipid-lowering drugs, which could be related to the otherwise low cardiovascular risk based on young age, although again there is a high risk of under-treatment.

An overview of this cohort compared to other published 22q11 DS cohorts is presented in Table 5.

**Table 5:** Prevalence of endocrinological manifestations in patients with 22q11 DS in other studies

Study (year) (ref)	*N*	HypoCa	HypoPT	HypoT	HyperT	Obesity
Ryan et al. (1997, ref 3)	290	60%	ND	ND	ND	ND
Taylor et al. (2003, ref 9)	61	30%	ND	ND	ND	ND
Choi et al. (2005, ref 17)	61	32%	13%	ND	1%	ND
Bassett et al. (2005, ref 26)	78	64%	ND	20%	5%	ND
Cheung et al. (2014, ref 8)	138	80%	63%	30%	ND	ND
Voll et al. (2017, ref 18)	202	ND	ND	19%	ND	43%
**Present study**	75	35%	15%	44%	8%	38%

*HypoCa* hypocalcemia, *HypoPT* hypoparathyroidism, *HypoT* hypothyroidism, *HyperT* hyperthyroidism, *Ref* reference, *ND* no data

Finally, we studied the adherence to the available guidelines in this real-world cohort. Guidelines on 22q11 DS propose at least a yearly determination of serum calcium and TSH and advise supplementary determination of serum calcium during periods of elevated stress [5, 30], which happened in a minority of our cohort. Guidelines also propose that all patients with 22q11 DS should take calcium and vitamin D supplements, independently of the diagnosis of hypoparathyroidism [5.30], which was the case in less than one-fifth of this real-world cohort, showing substantial under-treatment. This was particularly the case for the subgroup without hypoparathyroidism. According to the international guidelines on the management of chronic hypoparathyroidism [12, 14], at least yearly monitoring of serum calcium is warranted, as well as yearly evaluation of 24-h calciuria, 5-yearly renal imaging, and an ophthalmologic exam and brain imaging at baseline [12, 14]. In this cohort, half of the patients with confirmed hypoparathyroidism had a yearly calcium measurement. The other screening exams, when performed, were almost always performed for other indication than screening for chronic complications of hypoparathyroidism. Our findings underline the potential for improvement with regards to the adherence to international guidelines, which might insufficiently be known by non-endocrinologists.

Our study is not free from limitations. We present a single-center retrospective study. We lack data on ionized calcium measurements. Follow-up was not standardized but reflects current practice. The strength of this study consists of the large patient cohort and the combined evaluation of parathyroid, thyroid, and metabolic disease. In addition, our study was conducted in an adult population, compared to previous studies which are frequently performed in a pediatric population. We also evaluated the adherence to international guidelines for endocrinological follow-up of patients with 22q11 DS and propose a format for the endocrinological follow-up of patients with 22q11 DS (Table 6).

**Table 6:** Endocrinological follow-up in patients with 22q11 DS

**Parathyroid function**	Yearly screening of serum calcium/phosphate/PTH/albumin/Mg/creatinine/25OH vitamin D Extra measurement of calcium/albumin during elevated stress (e.g., neonatal period, infection, surgery) Daily supplementation of calcium (1000 mg and extra during stress and symptoms) and cholecalciferol, aiming at serum 25OHD > 20 µg/L In case of hypoparathyroidism treated with active vitamin D analog: . Minimum twice yearly follow-up of serum calcium/phosphate/PTH/albumin/Mg/creatinine/25OH vitamin D . Yearly follow-up of 24 h urine (creatinine/calcium/sodium) . Baseline and every 5 y: renal US . Baseline: CT brain . Baseline: Ophthalmological exam (cataract)
**Thyroid function**	Yearly screening of serum TSH Baseline: thyroid US
**Metabolic profile**	Yearly BMI Yearly blood pressure Yearly screening of fasting glucose/lipids/liver function

*BMI* body mass index, *CT* computed tomography, *Mg* magnesium, *PTH* parathyroid hormone *US* ultrasound, *TSH* thyroid stimulating hormone

## Conclusion

Hypoparathyroidism, hypothyroidism, and obesity are common endocrinological manifestations in patients with 22q11DS. However, hypoparathyroidism is probably frequently underdiagnosed and undertreated. Screening for endocrine manifestations in the 22q11DS is proposed by international guidelines, but adherence to these guidelines is rather low. Prospective registries are needed to have better estimates of the endocrine manifestations in patients with 22q11DS and the adherence to guidelines. Education of patients and general physicians on endocrine manifestations is important. We propose a format for the endocrinological follow-up of patients with 22q11 DS Multidisciplinary follow-up including an endocrinologist is important in 22q11DS patients, along with regular visits with the general physician.

## Data Availability

Additional data may reasonably be requested from the corresponding author.
